# Transcriptome Analysis Reveals the Potential Role of Long Non-coding RNAs in Mammary Gland of Yak During Lactation and Dry Period

**DOI:** 10.3389/fcell.2020.579708

**Published:** 2020-11-25

**Authors:** Xiaoyun Wu, Xuelan Zhou, Lin Xiong, Jie Pei, Xixi Yao, Chunnian Liang, Pengjia Bao, Min Chu, Xian Guo, Ping Yan

**Affiliations:** Key Lab of Yak Breeding Engineering, Lanzhou Institute of Husbandry and Pharmaceutical Sciences, Chinese Academy of Agricultural Sciences, Lanzhou, China

**Keywords:** lncRNA, lactation, mammary gland, RiboZero RNA-seq, yak

## Abstract

The mammary gland is a remarkably dynamic organ of milk synthesis and secretion, and it experiences drastic structural and metabolic changes during the transition from dry periods to lactation, which involves the expression and regulation of numerous genes and regulatory factors. Long non-coding RNA (lncRNA) has considered as a novel type of regulatory factors involved in a variety of biological processes. However, their role in the lactation cycle of yak is still poorly understood. To reveal the involved mechanism, Ribo-zero RNA sequencing was employed to profile the lncRNA transcriptome in mammary tissue samples from yak at two physiological stages, namely lactation (LP) and dry period (DP). Notably, 1,599 lncRNA transcripts were identified through four rigorous steps and filtered through protein-coding ability. A total of 59 lncRNAs showed significantly different expression between two stages. Accordingly, the results of qRT-PCR were consistent with that of the transcriptome data. Gene Ontology (GO) and Kyoto Encyclopedia of Genes and Genomes (KEGG) enrichment analyses indicated that target genes of differentially expressed lncRNAs (DELs) were involved in pathways related to lactation, such as ECM-receptor interaction, PI3K-Akt signaling pathway, biosynthesis of amino acids and focal adhesion etc. Finally, we constructed a lncRNA-gene regulatory network containing some well known candidate genes for milk yield and quality traits. This is the first study to demonstrate a global profile of lncRNA expression in the mammary gland of yak. These results contribute to a valuable resource for future genetic and molecular studies on improving milk yield and quality, and help us to gain a better understanding of the molecular mechanisms underlying lactogenesis and mammary gland development of yak.

## Introduction

In the past decade, human whole-genome transcriptional study discovered that approximately two-thirds of genomic DNA is pervasively transcribed (Djebali et al., 2012). Still less than 2% of the mammalian genome translates into the proteins, indicating that a considerable portion of the mammalian genome is transcribed into non-coding RNAs (ncRNAs) and do not function in protein-coding (Kopp and Mendell, 2018). The function-related non-protein-coding genome is particularly evident for a kind of small non-coding RNAs (ncRNAs) such as microRNAs (miRNAs), PIWI-interacting RNAs (piRNAs) and small interfering RNAs (siRNAs) (Pauli et al., 2011). Aside from small RNA, long non-coding RNAs (lncRNAs) have been widely concerned in recent years as potentially novel and pivotal regulators of biological process. LncRNAs are a heterogeneous group of non-protein-coding transcripts of greater than 200 nucleotides, which expression levels are usually lower than that of protein-coding genes (Hezroni et al., 2015; Ulitsky, 2016). Based on their position in the genome, lncRNAs can be categorized as long intergenic ncRNA (lincRNAs), enhancer RNAs (eRNAs), intronic lncRNAs, anti-sense lncRNAs and promoter-associated short RNAs (pasRNA) (Weikard et al., 2017). The regulatory roles of lncRNAs are gradually being revealed in a diverse spectrum of biological processes such as genomic imprinting (Sleutels et al., 2002), RNA splicing (Gonzalez et al., 2015), chromosome conformation (Rinn and Chang, 2012), epigenetic regulation (Vizoso and Esteller, 2012), transcriptional control (Salmena et al., 2011), and allosterically regulating enzymatic activity (Quinn and Chang, 2016).

With the increasing interest in lncRNA, a growing number of studies have discovered numerous lncRNAs in mammals, including *Homo sapiens* (Khalil et al., 2009), *Mus musculus* (Guttman et al., 2010), *Bos taurus* (Kern et al., 2018), *Bubalus bubalis* (Huang et al., 2019), and *Sus scrofa* (Gao et al., 2017). Recent studies have showed that several lncRNAs serve a role in the developing mammary gland and lactation. Pregnancy-induced non-coding RNA (*PINC*), a mammalian specific and alternatively spliced lncRNA earliest found in rat mammary gland, which has a function in regulating survival and cell cycle progression of mammary epithelial cells (Ginger et al., 2006). Increased expression of *PINC* in HC11 cells inhibits lactogenic differentiation, while suppressed expression of *PINC* could promote differentiation (Shore et al., 2012). LncRNA *Zfas1* is localized in the epithelial cells of the mammary gland duct and alveoli. Loss of *Zfas1* could promote the cell proliferation and differentiation in mammary epithelial cell line (Askarian-Amiri et al., 2011). Nuclear-enriched abundant transcript 1 (*Neat1*) has been reported to play a critical role in corpus luteum (Nakagawa et al., 2014) and placenta (Gremlich et al., 2014) development process. It also involves in mouse mammary gland morphogenesis and lactation. Knockout *Neat1* in mouse alveolar cells could decrease the rates of cell proliferation (Standaert et al., 2014). LncRNA X inactivate−specific transcript (*XIST*) plays a vital role in the X-inactivation process in female mammals (Chureau et al., 2011). Previous observation demonstrated that *XIST* inhibits cell viability and promotes apoptosis in bovine mammary alveolar cell−T under inflammatory conditions (Ma et al., 2019).

Yak (*Bos grunniens*) is a multipurpose livestock, providing milk, meat, wool and transportation for indigenous people on the Qinghai-Tibetan Plateau. Yak milk and milk products are the main part of the daily diets of Tibetan nomads and play a key role in the health maintenance of Tibetans in the hypoxic environment. Compared with milk of dairy cattle, yak milk contains a higher content of protein, antioxidant vitamins, specific enzymes, and biologically active fatty acids (Ding et al., 2013; Guo et al., 2014). However, industrialized production of yak milk production is limited by the low milk yield of yak (150–500 kg of fresh milk per lactation) (Li et al., 2011). Therefore, improving milk yield is one of the significant breeding objectives in the yak industry. The mammary gland is a crucial secretory gland for milk synthesis and secretion, which provides necessary nutrients for human and neonatal offspring (McManaman and Neville, 2003; Tong et al., 2017). Post-pubertal mammary gland experiences a cycle of cell proliferation, differentiation, de-differentiation and apoptosis during its lactation process that is under the regulation of distinct hormones and regulating genes (Hennighausen and Robinson, 2005; Watson and Khaled, 2008). Detection of expression and function of coding genes and ncRNAs at different lactation stages of post-pubertal mammary gland development is not only essential for investigating the molecular mechanism of lactation, but also for improving milk yield and quality. A previous transcriptome study has identified numerous genes related to lactogenesis, milk secretion, and mammary gland development of yak during lactation and dry period (Fan et al., 2018). However, biological function of lncRNA have not been systematically identified in mammary glands of yak during the different lactation stages. Here, comparative analysis of lncRNAs in yak mammary gland was performed at two physiology stages (lactation period and dry period) by constructing five RNA sequencing (RNA-seq) libraries and sequencing. Our study will provide precious resources for yak lncRNA studies and contribute to a better understanding of the regulatory mechanisms of lactation.

## Materials and Methods

### Animal and Sample Collection

Five healthy and mastitis-free female Ashidan yaks in their second parity were selected for this study. All individuals were freely grazed on natural pasture with free drinking at Datong yak farm of Qinghai Province. Due to the limitation of sampling condition, we only selected two yaks at peak lactation (LP; 120 days postpartum; non-pregnant period), and three yaks at the dry period (DP; calved in the previous year, non-pregnant period). After slaughter, the rear mammary gland of each yak was cut immediately, and alveolar tissue from the middle of the upper one-third of the gland was harvested and stored in liquid nitrogen. All of the experimental protocols and procedures were approved by Animal Administration and Ethics Committee of Lanzhou Institute of Husbandry and Pharmaceutical Sciences of CAAS (Permit No. SYXK-2014-0002).

### Total RNA Isolation

Total RNA was isolated from mammary gland tissues with the TRIzol reagent following the manufacturer’s protocol (Invitrogen, CA, United States). The quality and purity of total RNA were evaluated by a NanoPhotometer^®^ spectrophotometer (IMPLEN, CA, United States), and integrity was assessed by the RNA Nano 6000 Assay Kit of the Bioanalyzer 2100 system (Agilent Technologies, CA, United States). The 260/280 ratio for each sample was approximately 2.0, and the RNA integrity number (RIN) was greater than 8.0.

### Library Preparation and Sequencing

The same amount of total RNA (3 μg) from each sample was used to construct RNA-Sequencing library, and ribosomal RNA was depleted from total RNA by Epicentre Ribo-zero^TM^ rRNA Removal Kit (Epicentre, United States). Subsequently, sequencing libraries were separately generated using the rRNA-depleted RNA by NEBNext^®^ Ultra^TM^ Directional RNA Library Prep Kit for Illumina^®^ (NEB, United States) following the manufacturer’s instructions. The quality of all libraries was assessed on the Agilent Bioanalyzer 2100 system (Agilent Technologies, Santa Clara, CA, United States). Sequencing libraries were then sequenced on an Illumina Hiseq 2500 platform (Novogene Bioinformatics Institute, Beijing, China) to generate 125 bp paired-end reads. Quality control of RNA-seq reads was performed using FastQC (Andrews, 2012).

### Data Analysis

Raw data (raw reads) of fastq format were first filtered through in-house Perl scripts. In this step, clean reads were obtained by removal of the reads containing adapter molecules, reads containing unknown bases (>10%), and low quality reads (more than 50% of the bases had quality scores ≤10) from raw reads. Meanwhile, the Q30 and GC contents of the clean data were calculated. Genome sequence of yak (BosGru_v2.0) and annotation file were downloaded from NCBI^1^. Paired-end clean reads were mapped to the yak genome using Tophat2 (v2.0.9) (Kim et al., 2013). Scripture (beta2 version) (Guttman et al., 2010) and Cufflinks (v2.1.1) software (Trapnell et al., 2010) were used to assemble the transcripts. Cuffdiff (version 2.1.1) (Trapnell et al., 2010) was used to calculate thefragments per kilobase of exon per million fragments mapped (FPKM) value of transcripts with default parameters, including lncRNAs and mRNAs in each sample.

### LncRNAs Identification

**FIGURE 1:**
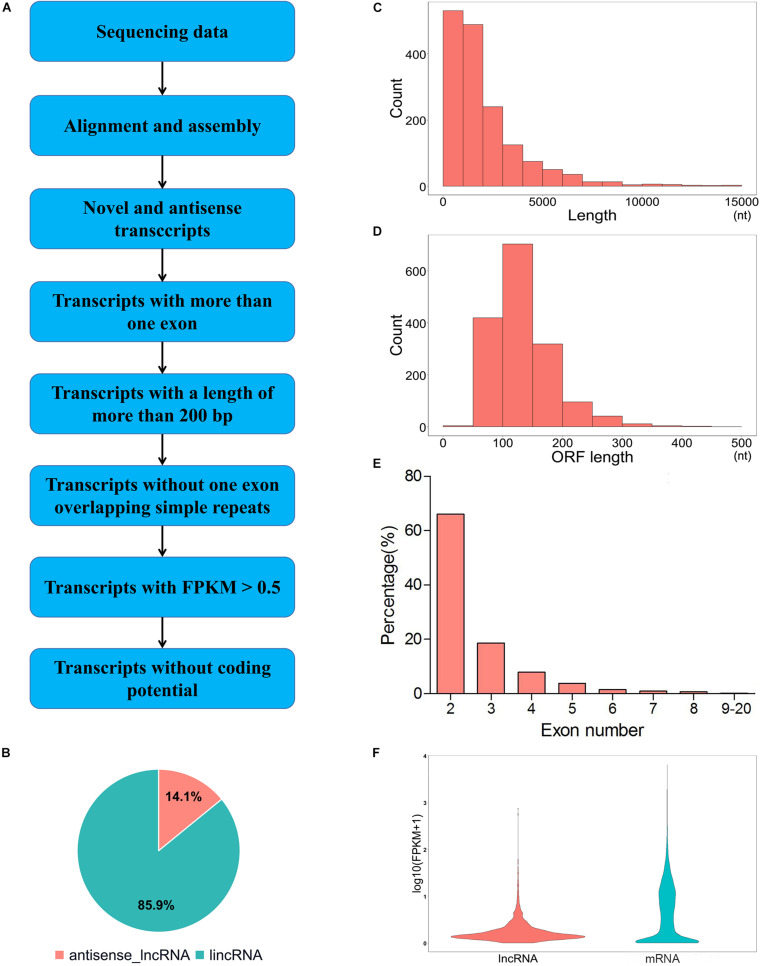
Generation and characterization of lncRNAs in mammary gland of yak. **(A)** Pipeline for identification of lncRNAs. **(B)** Classification of lncRNAs. **(C)** The length distribution of lncRNAs. **(D)** ORF length distribution of lncRNAs. **(E)** Exon number distribution of lncRNAs. **(F)** Expression level of lncRNAs and mRNAs. Red and blue represent lncRNAs and mRNAs, respectively.

To identify the putative lncRNA, we filtered the assembled transcripts through the following exclusion criterion (Figure 1A). (1) transcripts with a single exon were removed. (2) transcripts with a length of more than 200 nt were kept. (3) transcripts with FPKM ≥ 0.5 were kept. (4) transcripts that overlap with the exon region of the database annotation were removed. (5) CNCI (Coding-Non-Coding-Index, v2) (Sun et al., 2013), CPC (encoding potential calculator, 0.9-r2) (Kong et al., 2007) and Pfam Scan (v1.3) (Punta et al., 2012) were used to predict the protein-coding ability of the transcripts. Transcripts without protein-coding ability were selected as a candidate set of lncRNAs. The expression levels of mRNA and lncRNA were calculated based on assembled transcript files. The edgeR (exact test for negative binomial distribution) Bioconductor package (Robinson et al., 2010) in R software was used to identify differentially expressed lncRNAs (DELs) and differentially expressed genes (DEGs) between LP and DP groups. For biological replicates, genes or lncRNAs with | log2 (fold change)| ≥ 1 and *P*-value < 0.05 were considered as differentially expressed according to the previous study (Zheng et al., 2018).

### LncRNA Target Gene Prediction and Functional Enrichment Analysis

The *cis*- and *trans*-acting of lncRNAs can modulate the interactions with target genes. The *cis* role of lncRNAs is to interact with neighboring target genes. In this study, the protein-coding genes located in 100 kb upstream and downstream of DELs were searched as described previously (Gao et al., 2019). The *trans* role refers to lncRNAs that regulate other genes on different positions of the genome. To identify the *trans*-target genes of the DELs, the expressed correlation of DELs and DEGs was estimated with Pearson’s correlation coefficient (*r*) using the cor.test function in R software. Those DEGs with a *P* value <0.05 and | *r*-value| > 0.95 were considered as *trans*-target genes. To infer the function of the lncRNA, we used the G:Profile web tool (Raudvere et al., 2019) to implement gene ontology (GO) analyses of target genes. Kyoto Encyclopedia of Genes and Genomes (KEGG) enrichment analysis of lncRNA target genes was conducted using KOBAS 3.0 software (Xie et al., 2011) by using a hypergeometric test. Pathway with gene count >5 and *P*-value <0.05 were considered signifcantly enriched by target genes. Based on the enrichment analysis results, function and quantitative trait locus (QTL) information of the differentially expressed *cis*- and *trans*-target genes, DELs and target genes associated with milk traits and lactation were selected to construct the regulatory network. We used Cytoscape v.3.7.2 software (Cline et al., 2007) to visualize the regulatory networks.

### Verification of Sequencing Data Using qRT-PCR

To verify the accuracy of sequencing results, quantitative real-time PCR (qRT-PCR) method was used to detect the relative expression of ten randomly selected DELs. One microgram of total RNA from each sample was transcribed into cDNA using Transcriptor First Strand cDNA Synthesis Kit (Roche Diagnostics, Mannheim, Germany) following the manufacturer’s protocols. The lncRNAs expressions were then measured by qRT-PCR using the LightCycler 96 Real-Time PCR system (Roche Diagnostics, Mannheim, Germany). The 20 μL PCR reactions included 1 μL of diluted cDNA, 1 μL of forward primer (10 μM), 1 μL of reverse primer (10 μM), 10 μL of SYBR TB Green mix (TaKaRa, Dalian, China), and 7 μL of ddH_2_O. The PCR program for amplification was as follows: an initial denaturation step of 30 s at 95°C, and 45 cycles of 5 s at 95°C, and 30 s at 60°C. A final melting program ranging from 65°C to 97°C with an increments of 0.5°C and acquiring fluorescence after each step. The relative expression of DELs was analyzed using the 2^–ΔΔCt^ method (Silver et al., 2006) and normalized by hydroxymethylbilane synthase (*HMBS*) and tyrosine 3-monooxygenase/tryptophan 5-monooxygenase activation protein, zeta polypeptide (*YWHAZ*) (Wu et al., 2019).

## Results

### Overview of RNA-seq Data

In order to identify novel lncRNAs in mammary gland of yak, five cDNA libraries were sequenced using Illumina Hiseq 2500 platform, and 503.07 and 299.23 million raw paired-end reads were obtained in lactation and dry stages, respectively. After removing the adaptor sequence and low-quality raw reads, we obtained 784.05 million clean reads with an average of 94.35 million reads (ranging from 135.20 to 181.06) for each sample. The GC contents were 50.51–53.23% while the Q30 ranged between 96.35 and 96.53%. Of the clean reads, 83.79–86.48% could be perfectly mapped to the yak reference genome by the Tophat2 software (Kim et al., 2013) (Table 1). Pearson’s correlation analysis showed that the correlation between the groups was strong and providing reliable data for the next steps (Supplementary Figure 1).

**TABLE 1:** Summary of RNA-Seq data and mapping.

Sample name	Raw reads	Clean reads	Q30 (%)	GC content (%)	Total mapped	Uniquely mapped
DP1	184408224	181065646	96.5	53.07	156206414 (86.27%)	143152662 (79.06%)
DP2	155782622	152534698	96.53	53.23	131660504 (86.32%)	120711885 (79.14%)
DP3	162876662	158253090	96.35	53.07	132600127 (83.79%)	121998364 (77.09%)
LP1	160155930	156985398	96.53	53.17	135755839 (86.48%)	122863727 (78.26%)
LP2	139072986	135208918	96.35	50.51	115961807 (85.76%)	107999673 (79.88%)

### Characterization and Expression Profiling of LncRNAs in the Yak Mammary Gland

**FIGURE 2:**
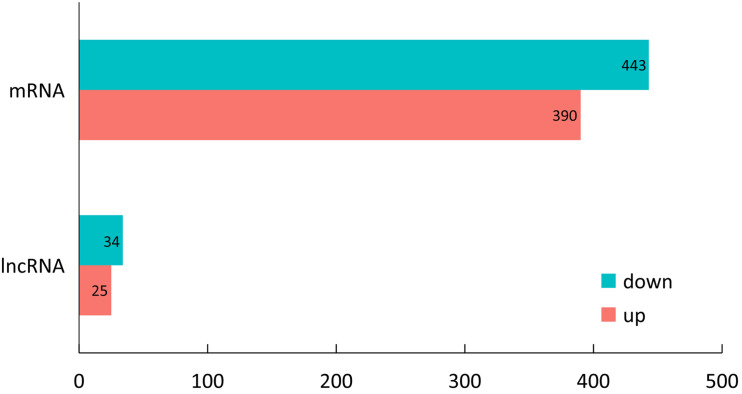
Number of upregulated and downregulated DEGs and DELs of LP vs DP.

We identified 1,599 novel lncRNAs from the yak mammary gland tissues by using CNCI, CPC and Pfam software (Supplementary Table 1 and Figure 2). According to the genome alignment, these lncRNA transcripts were classified into 1,374 (85.9%) lincRNA and 225 (14.1%) anti-sense lncRNA (Figure 1B). The size of lncRNAs length in the mammary gland ranged from 219 to 26,695 nt, with 63.7% of the total number of lncRNAs were approximately 200 to 2,000 nt in length (Figure 1C). The lncRNAs whose ORF length ranged from 101 to 150 bp occupied the largest proportion of the total lncRNAs (Figure 1D). Most of the lncRNAs contained three or fewer exons (84.6%) and the average exon number of the lncRNA was 2.65 (Figure 1E). Expression levels of the lncRNAs were also lower than those of the genes (Figure 1F).

Expression levels of the lncRNA transcripts were determined through FPKM method by using Cufflinks (v2.1.1) software. A total of 59 DELs were identified transcripts between LP and DP groups (| log2 (fold change)| ≥ 1 and *P*-value < 0.05). Of these DELs, 25 lncRNAs were significantly upregulated and 34 lncRNAs were downregulated in the mammary gland at lactation stage (Figure 2 and Supplementary Table 2). We also found 833 DEG between LP and DP groups | log2 (fold change)| ≥ 1 and *P*-value < 0.05) (Figure 2 and Supplementary Table 3).

### LncRNAs Target Genes of Cis and Trans Regulated

To further explore the function of lncRNAs in yak mammary gland, we identified potential *cis*- and *trans*-target genes of DELs, and predicted the function of target genes through GO and KEGG pathways enrichment analysis. The *cis*-targets of lncRNAs were predicted by screening genes around 100 kb upstream and downstream of lncRNAs. The results showed that there were 42 lncRNAs correspond to 130 *cis*-target genes (Supplementary Table 4). GO enrichment analysis revealed *cis*-target genes of DELs were significantly enriched in 182 terms (131 under biological processes, 15 under cellular compartments, and 36 under molecular functions) (adjusted *P* value <0.05). Detailed information is listed in Supplementary Table 5. The most significantly enriched GO terms were cellular anatomical entity, cellular anatomical entity, binding, biological regulation and intracellular. However, there was no significant enrichment of pathway for *cis*-target genes of DELs.

**FIGURE 3:**
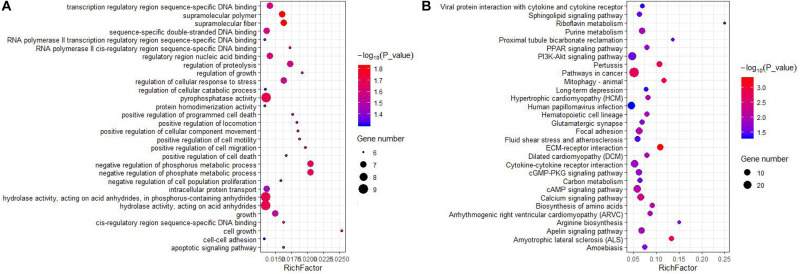
GO and KEGG enrichment of *trans*-target genes of DELs. **(A)** Top30 GO enrichment terms. **(B)** Top30 KEGG pathway enrichment terms. The horizontal axis represents a rich factor and the vertical axis represents the pathway. The size of the bubble indicates the number of target genes enriched in the pathway, and the color of the bubble represents a different *P*-value range. Rich Factor is the ratio of differentially expressed gene numbers annotated in this pathway terms to all gene numbers annotated in this pathway term.

We also predicted potential target genes of lncRNAs in a *trans* manner. A total of 3,228 interactions (including 2,549 positive and 679 negative correlations) were identified between 59 DELs and 758 DEGs (Supplementary Table 6). In our results, 23 *cis*-genes overlapped with *trans*-target genes. GO analysis showed that the *trans*-target genes were significantly enriched in 836 terms (654 biological processes, 88 cellular compartments, and 94 molecular functions), which involves a variety of biological processes (Supplementary Table 7). The top enriched terms were cellular process, cellular anatomical entity and bingding (Figure 3A). It is important that some of the terms were related to mammary gland development, such as cell surface receptor signaling pathway, cell differentiation, cell population proliferation, epithelial cell proliferation, and mammary gland development. KEGG analysis revealed the *trans*-target genes were enriched in 35 pathways (Supplementary Table 8). The top 30 KEGG pathways are showed in Figure 3B. Importantly, a few pathways were associated with lactation, such as ECM-receptor interaction, PI3K-Akt signaling pathway, PPAR signaling pathway, biosynthesis of amino acids, suggesting that lncRNAs act in the *trans*-regulation of genes related to lactation. Moreover, multiple DELs had a common target gene.

### Integrated Analysis

**FIGURE 4:**
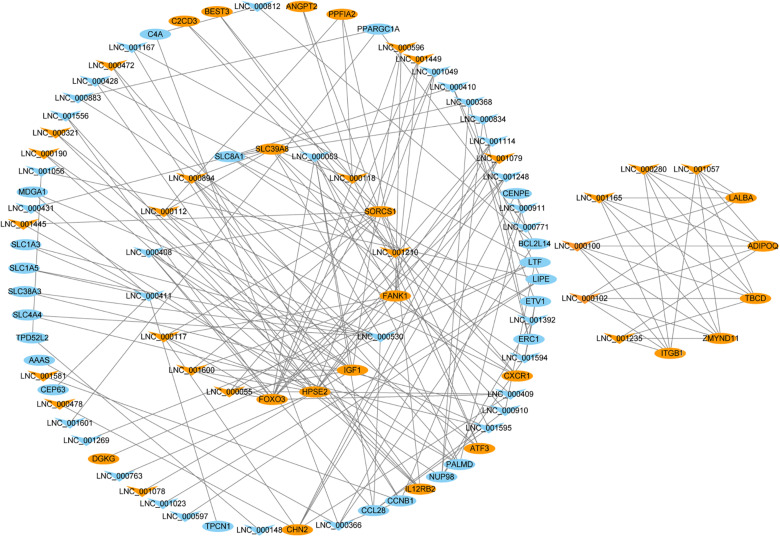
Network plot of candidate lncRNAs and mRNAs. The orange triangles, blue triangles, orange round and blue round represent upregulated lncRNAs, downregulated lncRNAs, upregulated mRNAs and downregulated mRNAs, respectively.

To better understand the relationship between DELs and lactation process, we chose 42 target genes related to lactation and mammary gland development to construct lncRNA-gene regulatory network. The regulatory network showed that 54 DELs interacted with at one target genes associated with lactation and mammary gland development (Figure 4 and Supplementary Table 9). Notably, multiple lncRNAs (LNC_001595, LNC_000894 and LNC_001600 and others) appear to regulate insulin like growth factor 1 (*IGF1*), indicating that they might regulate lactation process though the PI3K-Akt pathway.

### Verification of Gene Expression Profiles Using qRT-PCR

**FIGURE 5:**
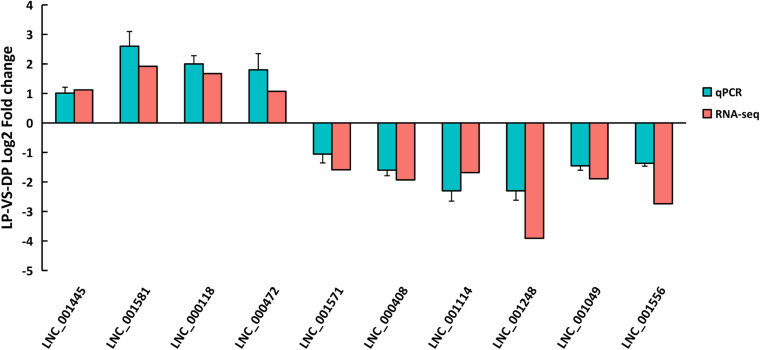
Validation and comparison of log2(fold change) in ten DELs between qRT-PCR and RNA-Seq.

To validate the reproducibility of the DELs obtained from RNA-seq, ten DELs were randomly selected for confirmation by qRT-PCR. The primer details are presented in Supplementary Table 10. As showed in Figure 5, the expressions of LNC_001445, LNC_001581, LNC_000118 and LNC_000472 were upregulated, whereas LNC_001571, LNC_000408, LNC_001114, LNC_001248, LNC_001049 and LNC_001556 were downregulated in LP compared to DP. All ten DELs had similar expression patterns in comparison to the RNA-seq data, indicating the reliability of our RNA-seq data.

## Discussion

The mammary gland is a complex organ for synthesis and secretion of milk, which undergoes functional differentiation during the female reproductive cycle (Hurley, 1989). During pregnancy and lactation, epithelial cells of the mammary gland start to proliferate and differentiate into functional alveolar cells, which produce and secrete milk after parturition (Macias and Hinck, 2012). In the dry period, apoptosis of the majority of differentiated alveolar cells was induced, and gland restores to a pre-pregnancy state (Watson, 2006). Deciphering these physiological changes is essential to explore the complex molecular mechanisms underlying the transition from the dry period to lactation. One of the important aspects was the transcript originated from the non-coding regions of the genome. However, systematic prediction and regulatory function of lncRNAs in yak mammary gland have rarely been reported. In this study, we comprehensively investigated lncRNAs in the mammary gland of yak at lactation and dry period.

In the present study, more than 83.79% of the clean reads were mapped to the yak genome, which was similar to those of other RNA-seq studies (Zi et al., 2018). Similar to lncRNA features of previous studies, the average spanning exon number and expression levels of lncRNA was much less than that of the genes, indicating the reliability of lncRNA identification (Zheng et al., 2018; Li et al., 2020). Furthermore, we identified a total of 59 DELs from 5 samples, comprising 25 upregulated and 34 downregulated DELs in LP compared with the DP (| fold change| > 2, *P*-value <0.05). Remarkably, in our study design, only two biological replicates in LP were used due to the limited sample availability. Rapaport et al. (2013) demonstrated that over 90% of differently expressed genes at the top expression levels could be detected with using two replicates and 5% of the reads. However, higher biological replicate numbers are still recommended to improve the detection power.

Recent studies have demonstrated that lncRNAs control gene expression through both *cis*- and *trans*-acting mechanism, which play an important role in a wide range of biological processes (Bhat et al., 2016). From this view, the function of lncRNA can be inferred on the basis of knowledge of their target genes. *Cis*-regulatory lncRNAs have enhancer-like activity and promote expression of neighboring genes. Accordingly, the *cis* prediction analysis showed that there were 42 DELs near to 130 genes with less than 100 kb distance. It is worth noting that some of the *cis*-target genes were involved in lactation and mammary gland development. For example, AKT Serine/Threonine Kinase 1 (AKT1) was predicted to be a target gene of LNC_000763, which regulates of milk synthesis and metabolism in the lactating mammary gland (Boxer et al., 2006). Constitutively active Akt1 in mice delayed mammary gland involution and epithelial cell apoptosis (Ackler et al., 2002). In contrast, depletion of Akt1 in mice resulted in a few mammary gland developmental defects, such as ductal outgrowth and deficiency in terminal end bud growth and alveolar bud development (LaRocca et al., 2011). LIM homeobox 3(*LHX3*) is a predicted *cis*-target of LNC_000108, which regulates the release of hormones related to lactation and metabolism (Pfäffle and Klammt, 2011). Liu et al. (2011) found that variations of *LHX3* gene were significantly associated with milk performance in goat. Secreted frizzled-related protein-2 (*SFRP2*) was predicted to be a target gene of LNC_001049. A previous study indicated that the interaction of SFRP2 with extracellular matrix (ECM) could prevent apoptosis of mammary epithelial cells (Lee et al., 2004). LNC_000118 was predicted to act on the target gene activating transcription factor 3 (*ATF3*). It is reported that the expression of ATF3 was significantly different in the mammary glands of Holstein cows with extremely high and low content of milk protein and fat (Cui et al., 2014). During the lactation cycle, the expression of ATF3 increased to a peak at day 15, followed by a decrease through day 240 relative to parturition in bovine mammary tissue (Invernizzi et al., 2012). These findings suggest that lncRNA might regulate lactation, milk synthesis and mammary gland development through their actions on neighboring genes.

Still, many lncRNAs act in *trans* mode to regulate the target genes, which are distant from the transcription sites of the lncRNAs. Co-expression analysis identified 59 DELs were interacted with 758 genes based on the expression correlation coefficient (*r-*value > 0.95 or < −0.95 and *P*-value <0.05). The results of KEGG analysis of the *trans*-target genes were involved in several keys signaling pathways, such as ECM-receptor interaction, PI3K-Akt signaling pathway, biosynthesis of amino acids and focal adhesion. These above KEGG pathways have been suggested to participate in the lactation process and mammary gland development (Watson, 2006; Nagy et al., 2007; Kim and Wu, 2009; Macias and Hinck, 2012). PPARG Coactivator 1 Alpha (*PPARGC1A*) is predicted to be a target gene of LNC_000883, LNC_001601 and LNC_000771. PPARGC1A, also known as PGC-1a, is a transcriptional coactivator that regulates genes involved in eglucose and lipid transportation and oxidation, which plays a crucial role in mammary gland development and lactation (Qiu et al., 2020). The polymorphism of the *PPARGC1A* gene has been proved to be significantly associated with milk fat yield, milk fat composition, milk protein percentage (Weikard et al., 2005; Khatib et al., 2007; Schennink et al., 2009). Ten DELs (such as LNC_001595, LNC_000894 and LNC_001600) were co-expressed with insulin like growth factor 1 (*IGF1*), a master regulator of mammary gland development, which considered as a candidate gene for milk protein and fat yields in Polish Holstein Friesian cows (Szewczuk et al., 2011). It is also reported that overexpression of *IGF1* gene in mice during pregnancy and lactation caused a delay of the apoptotic loss of mammary epithelial cells during the declining phase of lactation (Hadsell et al., 2002). In addition, 6 DELs were predicted to act on the target gene beta1 integrin (*ITGB1*), which plays a crucial role in the maintenance of functional mammary stem cells and mammary morphogenesis (Taddei et al., 2008). In the mammary luminal cell population, conditional deletion of *ITGB1* could inhibit the alveologenesis and lactation (Naylor et al., 2005). Furthermore, our results showed that a cluster of DELs regulates well-known genes affecting milk traits; e.g., lactotransferrin (*LTF*) (El-Domany et al., 2019) and lactalbumin alpha (*LALBA*) (García-Gámez et al., 2012). These observations suggested that these lncRNAs could be closely related to milk secretion due to their trans-targeted genes affecting lactation and mammary gland development. Although these lncRNAs need further experimental investigation, this information may help us to explore the potential regulatory mechanisms of lncRNA involved in lactation and mammary gland development in the yak.

## Conclusion

In the current study, we analyzed lncRNA expression profiles in the mammary gland of yak at two different physiological stages (LP and DP) for the first time. A total of 59 DELs were identified between two stages. Furthermore, several DELs related to lactation and mammary gland were annotated based on GO and KEGG analysis of *cis*- and *trans*-target genes. The DELs in the regulatory network may be considered as promising targets for the exploration of functional lncRNAs in yak and thus could be used to improve the milk traits. Considering the fact that the lncRNAs annotated on yaks are not complete, this work provides a valuable resource for improving the lncRNA database of yak and ideal candidates for future studies to illustrate the molecular mechanisms linking lncRNA to the regulation of lactation and mammary gland development.

## Data Availability Statement

The datasets generated for this study can be found in the Sequence Read Archive (https://www.ncbi.nlm.nih.gov/sra) at NCBI, with the BioProject ID: PRJNA626061.

## Ethics Statement

The animal study was reviewed and approved by Animal Administration and Ethics Committee of Lanzhou Institute of Husbandry and Pharmaceutical Sciences of CAAS (Permit No. SYXK-2014-0002).

## Author Contributions

XW, PY, and XG conceived the research and drafted the manuscript with comments from all authors. XW, XZ, JP, LX, and XY conducted the experiments and analyses. CL, PB, and MC participated in experiments and revised the manuscript. All authors approved the final version.

## Conflict of Interest

The authors declare that the research was conducted in the absence of any commercial or financial relationships that could be construed as a potential conflict of interest.

## Footnotes

1 http://www.ncbi.nlm.nih.gov/

## Supplementary Material

Supplementary Figure 1 Pearson’s correlation analysis between samples.

Supplementary Figure 2 Candidate lncRNA identification with coding potential assessment of CNCI, CPC2, and PfamScan.
