# Supplementary figures and images for: Transcriptome Analysis Reveals the Potential Role of Long Non-coding RNAs in Mammary Gland of Yak During Lactation and Dry Period
Source: Front Cell Dev Biol. 2020 Nov 25;8:579708. doi: 10.3389/fcell.2020.579708 (PMC7723986; doi:10.3389/fcell.2020.579708)

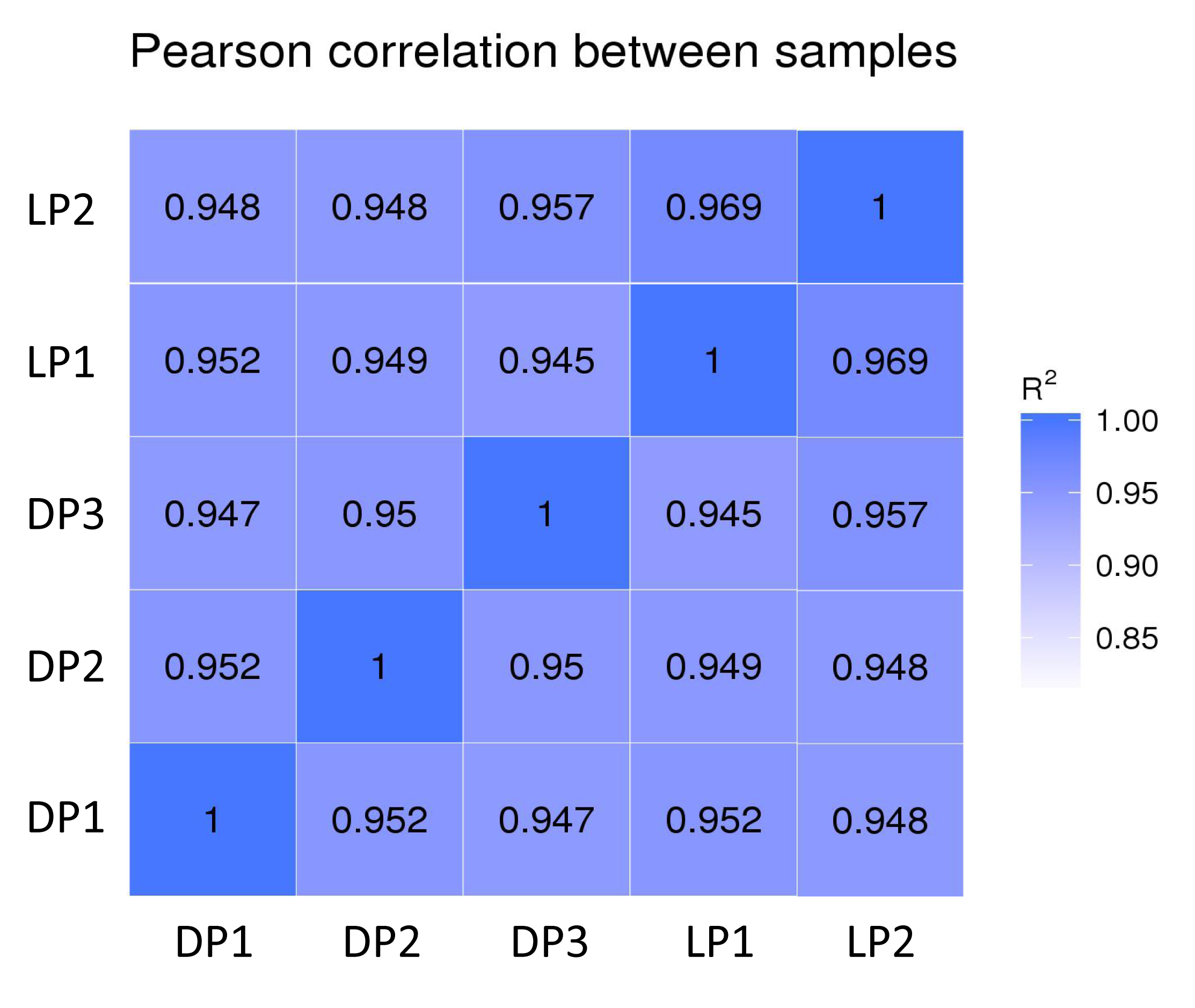

Supplement: Supplementary Figure 1 — Pearson’s correlation analysis between samples. [file Image_1.TIF]

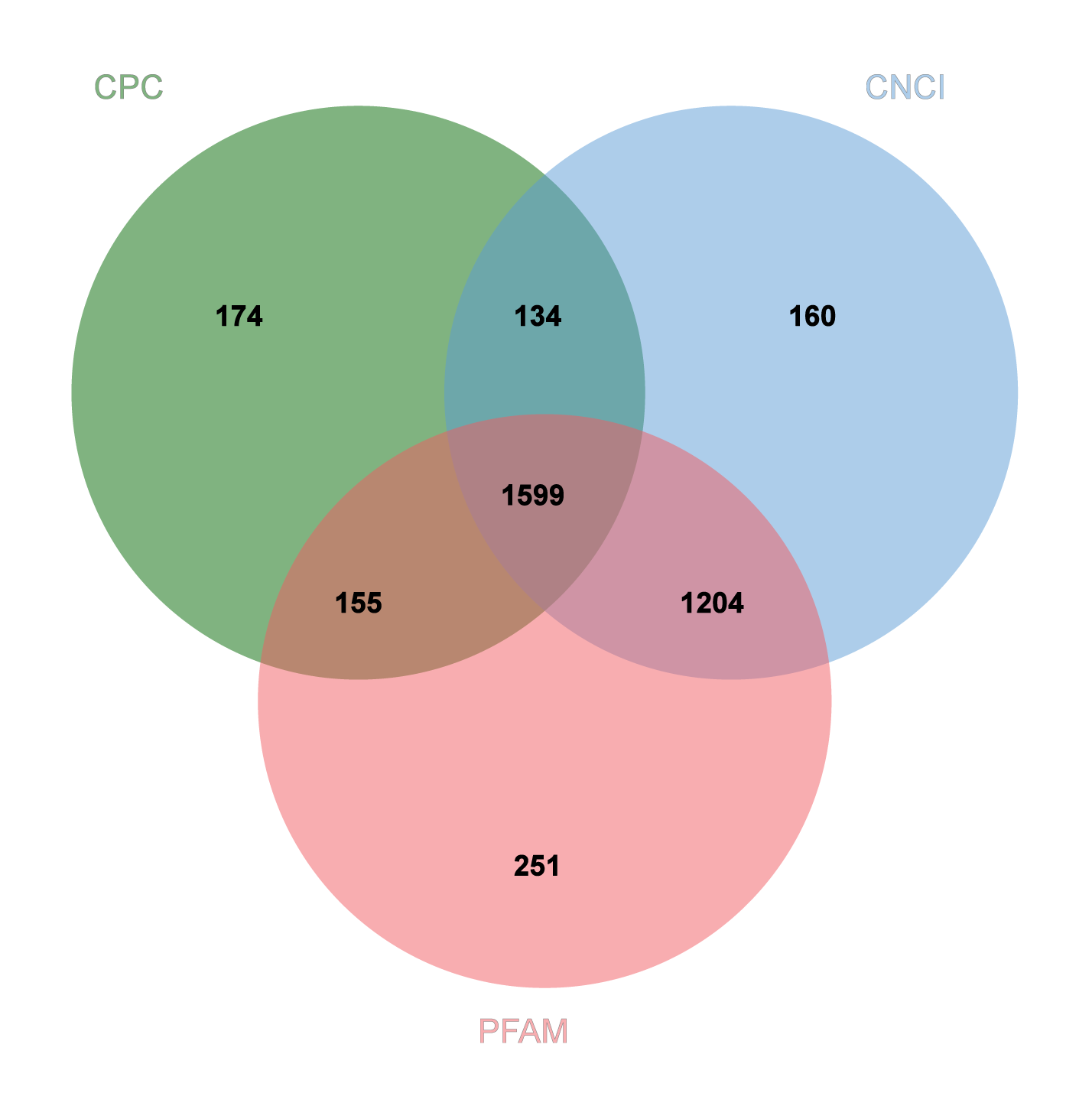

Supplement: Supplementary Figure 2 — Candidate lncRNA identification with coding potential assessment of CNCI, CPC2, and PfamScan. [file Image_2.TIF]
